# Loss of *Myostatin* Affects m6A Modification but Not Semen Characteristics in Bull Spermatozoa

**DOI:** 10.3390/ijms26020591

**Published:** 2025-01-12

**Authors:** Chao Hai, Linfeng Wang, Song Wang, Anqi Di, Lishuang Song, Xuefei Liu, Chunling Bai, Guanghua Su, Lei Yang, Guangpeng Li

**Affiliations:** State Key Laboratory of Reproductive Regulation and Breeding of Grassland Livestock, College of Life Science, Inner Mongolia University, Hohhot 010070, China; h15248037201@163.com (C.H.); linwang202312@163.com (L.W.); wangsong199852@163.com (S.W.); anqi_di@126.com (A.D.); xiaoshuang2000@126.com (L.S.); liuxuefei1006@126.com (X.L.); chunling1980_0@163.com (C.B.); suguanghua0707@163.com (G.S.)

**Keywords:** *Myostatin*, gene-edited cattle, sperm, m6A, *RHOA*

## Abstract

N6-methyladenosine (m6A) modification is a key methylation modification involved in reproductive processes. *Myostatin* gene editing (MT) in cattle is known to enhance muscle mass and productivity. However, the changes in m6A modification in MT bull sperm remain poorly understood. In the MT and wild-type (WT) groups, we identified 25,542 and 22,253 m6A peaks, respectively, mainly concentrated in the coding sequence (CDS) and 3′ untranslated region (UTR) of genes. The MT group showed an increase in gene transcription, but there was no significant difference in the overall m6A peaks pattern. There was also no significant difference in m6A motif and chromosome distribution between MT and WT groups. Most genes had less m6A modification sites. A total of 1120 m6A peaks were significantly different, corresponding to 1053 differentially m6A-methylated genes (DMMGs). These DMMGs are mainly associated with G protein-coupled receptor signaling pathways and the overall composition of the cell membrane. Furthermore, an MCL clustering analysis of 111 differentially m6A-methylated and expressed genes identified seven key genes (*RHOA*, *DAAM1*, *EXOC4*, *GNA12*, *PRICKLE1*, *SCN1A,* and *STXBP5L*), with the cytoskeleton and migration-related gene, *RHOA*, being the most important gene located at the center of the gene network. However, the analysis of sperm morphology and motility indicated no significant changes in semen volume, sperm count, sperm viability, plasma membrane integrity, acrosome membrane integrity, or mitochondrial membrane integrity. This study provides a map of m6A methylation in spermatozoa from MT and WT bulls, identifies key differential m6A genes that are affected by the myostatin gene but do not affect sperm morphology and viability in MT bulls, and provides a theoretical basis for the breeding quality of MT bulls.

## 1. Introduction

Myostatin is a member of the transforming growth factor-β (TGF-β) family of proteins [1]. It has been identified as a key regulator of muscle growth and development in various species, including bovines [2]. Mutations in the *Myostatin* gene lead to increased skeletal muscle mass and decreased body fat, thereby enhancing the efficiency of meat production in cattle [3,4]. Compared with those in control sperm, the knockout of the *Myostatin* gene in boars has been shown to result in no significant differences in sperm concentration, motility, plasma membrane integrity, aberration rate, acrosomal integrity, or mitochondrial activity [5]. In sheep, the double knockout of the *Myostatin* and fibroblast growth factor 5 (*FGF5*) genes resulted in normal spermatozoa structure and all sperm parameters and no significant differences in fertilization rates [6]. However, studies in cattle are incomplete, as there is limited information regarding the impact of *Myostatin* knockout on sperm characteristics, fertilization capacity, and subsequent reproductive performance. Further research is needed to determine whether *Myostatin* gene editing affects these parameters in cattle and to establish whether such edits have any implications for fertility and productivity.

N6-methyladenosine (m6A) modification is a key regulatory mechanism in many biological processes [7]. In skeletal muscle, m6A plays a significant role in modifying enzymes and regulating key mechanisms. Emerging evidence highlights its pivotal functions in controlling gene expression during skeletal muscle development, including roles in myogenic differentiation, muscle regeneration, and muscle hypertrophy [8]. For instance, the m6A methyltransferase METTL3 has been shown to regulate m6A levels, influencing skeletal muscle size [9]. Studies on Liaoyu white cattle (LYWC), a local breed in Liaoning Province, China, known for rapid growth, high meat quality, and stress resistance, have revealed breed-specific m6A modification patterns. Compared with Simmental cattle, LYWC exhibited distinct m6A-modified genes involved in muscle contraction, fatty acid metabolism, and steroid biosynthesis pathways, which are crucial for muscle growth. The integration of m6A-seq and RNA-seq identified 316 genes with both m6A modification and differential expression, highlighting their role in muscle hypertrophy and productivity [10]. This may be related to m6A-associated genes in cattle, such as an increase in demethylases (FTO, ALKBH5) and a decrease in m6A methyltransferases (METTL3, METTL14, and WTAP) [11].

Differences in paternal sperm RNA and methylation can be transmitted to offspring and play an important role in offspring development [12,13]. Among these, m6A modification plays a crucial role in regulating the transcriptional and translational activity of genes essential for spermatogenesis [7,14]. In the testis, the primary organ of male reproduction, m6A plays a dynamic role in post-transcriptional regulation during spermatogenesis. Recent studies using MeRIP-Seq analysis have identified 8774 m6A peaks and 6206 m6A-modified genes across nine bovine testes at three developmental stages (pre-puberty, puberty, and post-puberty). Differential analysis revealed 502 genes with concurrent changes in mRNA expression and m6A modification, including key regulatory genes such as PLK4, PTEN, EGR1, and PSME4, which are implicated in testis development and spermatogenesis [15]. m6A modification in the 3′ UTR of mRNA has been shown to regulate the stability and translation of genes involved in sperm motility and acrosome formation [16]. Additionally, m6A modification of RNA-binding proteins has been implicated in the regulation of RNA splicing and translation [17].

However, the effect of m6A modification on the mRNA of *Myostatin* gene-edited (MT) bull sperm is currently unknown. Therefore, this study attempted to explain the differences in mRNA m6A methylation in sperm by comparing the semen quality and sperm morphology of *Myostatin* gene-edited and wild-type (WT, cattle that have not undergone gene editing) bulls. Our findings could further our understanding of the underlying mechanisms of *Myostatin* on reproduction in cattle and provide insights into improving cattle breeding programs for enhanced reproductive performance.

## 2. Results

### 2.1. Overall Analysis of the m6A Modification Pattern

**Figure 1:**
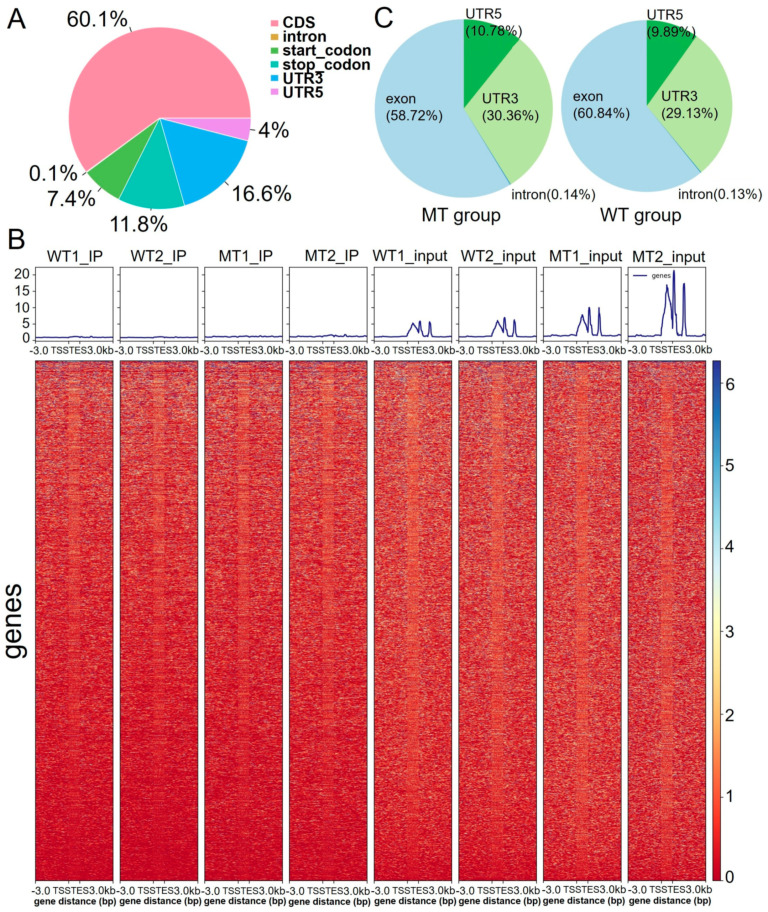
Overall distribution of m6A methylation. (**A**) Distribution of gene m6A peaks. (**B**) The enrichment of reads near the TSS and TES at the transcriptome initiation site of the gene. (**C**) Pie charts showing the percentage of m6A peaks in different groups.

To comprehensively understand the developmental characteristics of RNA transcripts in MT bull sperm compared to those in WT bull sperm, we conducted a comprehensive analysis of m6A modification patterns in the RNA transcripts of the MT group and WT group using MeRIP-Seq technology. A total of 25,542 and 22,253 m6A peaks were identified in the MT and WT groups. First, we examined the overall distribution of m6A modifications and found that m6A methylated sites were located mainly in the CDS regions (60.1%) and 3′ UTR (16.6%) of genes (Figure 1A).

A further analysis of the IP and input samples from the MT group indicated an increase in gene transcription, but the overall m6A peak pattern appeared similar (Figure 1B). A direct comparison of m6A distribution between the MT and WT groups similarly indicated comparable modification patterns (Figure 1C). These findings suggest that while transcriptional activity differs between MT and WT bull sperm, the global m6A modification landscape remains largely conserved.

### 2.2. Distribution of Differentially m6A-Methylated Sites

**Figure 2:**
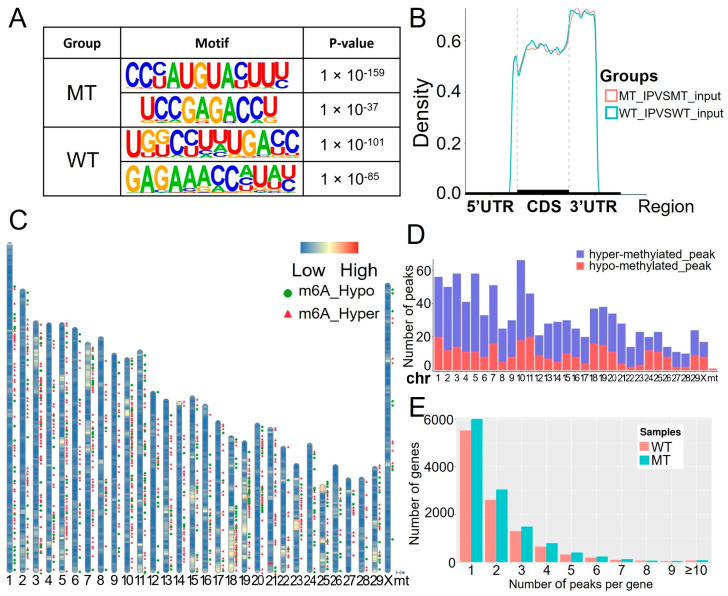
Distribution of m6A methylation across different groups. (**A**) Motifs enriched from m6A peaks were identified among the MT and WT groups. (**B**) Metagene plots displaying the regions of m6A peaks identified across the transcripts in the MT and WT groups. (**C**) Chromosomal distribution of all DMMSs within mRNAs. (**D**) Relative occupancy of DMMSs on each chromosome. (**E**) Number of m6A peaks per gene.

To delve deeper into the m6A modification profiles, we employed various bioinformatics tools, including the HOMER software suite and motif comparison algorithms, to identify and compare m6A motifs between the MT and WT groups. Our analysis indicated that the m6A motifs were similar between the two groups. Both groups prominently exhibited the well-known RRACH motif sequence, confirming its consistent role in m6A methylation (Figure 2A).

We then analyzed the distribution of m6A modifications across different genomic regions and observed that the modifications in both the MT and WT groups were predominantly enriched in the 3′ UTR (Figure 2B). To further investigate the spatial patterns of differentially m6A-methylated sites (DMMSs), we mapped these sites to chromosomes. The top five chromosomes with the highest abundance of DMMSs were ranked as 10, 3, 5, 1, and 7, revealing specific chromosomal hotspots for m6A modifications (Figure 2C,D).

Additionally, we examined the frequency of m6A modifications across genes. The results demonstrated a decreasing trend in the number of genes as the count of m6A peaks per gene increased, suggesting variability in methylation patterns across different transcripts (Figure 2E). This comprehensive analysis highlights both the conserved nature of m6A motifs and regional differences in modification patterns, providing deeper insights into the epitranscriptomic landscape of MT and WT bull sperm.

### 2.3. Functional Analysis of m6A Methylated Genes

**Figure 3:**
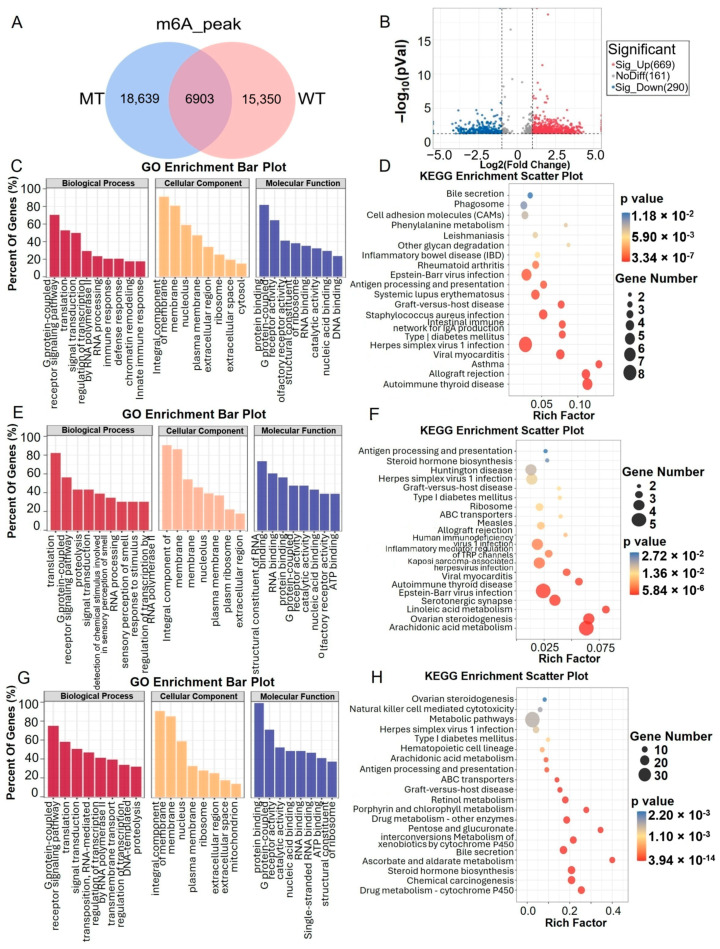
GO and KEGG analyses of differentially methylated m6A peaks. (**A**) Venn diagram of common m6A peaks within mRNAs in the MT and WT groups. (**B**) Volcano plot of significant m6A peaks. (**C**,**D**) GO and KEGG enrichment analysis of DMMGs specific to the MT group. (**E**,**F**) GO and KEGG enrichment analyses of DMMGs specific to the MT group. (**G**,**H**) GO and KEGG analyses of DMMGs common to the MT and WT groups.

The MT group exhibited 18,639 unique m6A peaks, while the WT group showed 15,350 unique m6A peaks. The MT and WT groups shared 6903 m6A peaks (Figure 3A). Of these, 1120 m6A peaks were significantly different, corresponding to 1053 differentially m6A-methylated genes (DMMGs, *p* < 0.05). The volcano plot showed 671 significantly upregulated peaks (corresponding to 644 DMMGs) and 290 significantly downregulated peaks (corresponding to 286 DMMGs) between the MT group and the WT group (|log_2_Fold change| ≥ 1, *p* < 0.05) (Figure 3B).

The differentially enriched biological processes for the unique m6A methylated genes and DMMGs in both groups included the G protein-coupled receptor signaling pathway and translation. In terms of cellular components, the major differences were observed in the integral components of the membrane (Figure 3C,E,G). In the MT group, the unique m6A methylation peaks were enriched in KEGG pathways related to diseases such as autoimmune thyroid disease, allograft rejection, and asthma (Figure 3D). The unique m6A methylation peaks in the WT group were also enriched in the autoimmune thyroid disease pathway, as well as arachidonic acid metabolism and linoleic acid metabolism (Figure 3F). The DMMGs were enriched in KEGG pathways such as drug metabolism—cytochrome P450 and steroid hormone biosynthesis (Figure 3H).

An analysis of the unique m6A-methylated genes and DMMGs’ associated enrichment pathways did not enrich for direct sperm functional pathways, suggesting that the effects of m6A-methylated genes on bull sperm are potentially embodied in the G protein-coupled receptor signaling pathway, which may play a role in hormone regulation and gene expression [18]. These results reveal a specific functional pattern of m6A methylation in MT bull spermatozoa, highlighting their potential role in cellular processes.

### 2.4. Combined Analysis of Differentially m6A-Methylated and Expressed Genes

**Figure 4:**
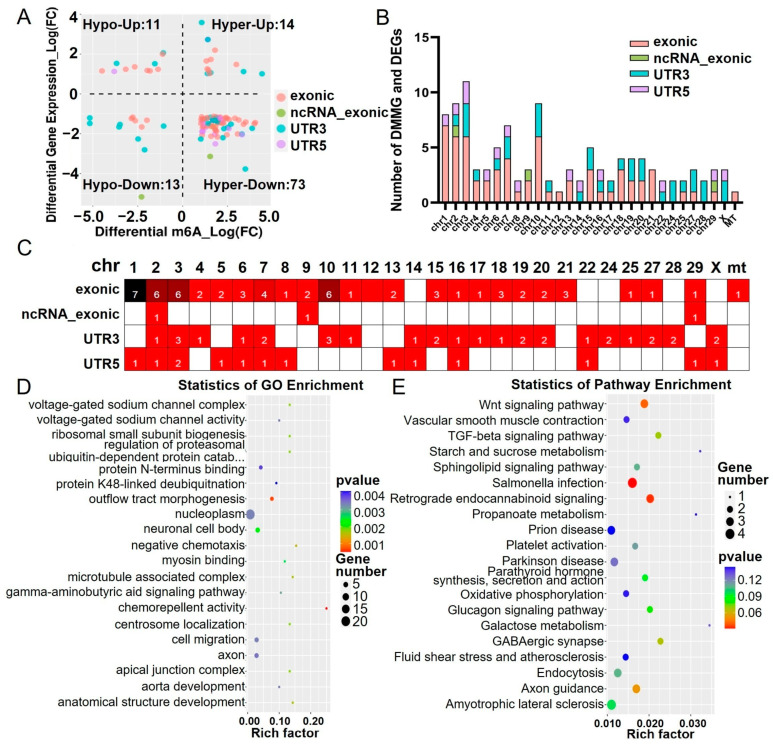
Association analysis between transcriptome-seq data and m6A-seq data. (**A**) Four-quadrant diagram of DMGs. Hyper-up represents upregulation of the m6A peak and upregulation of mRNA expression. Hyper-down regulation represents upregulation of the m6A peak and downregulation of mRNA expression. Hypo-up represents downregulation of the m6A peak and upregulation of mRNA expression. Hypo-down represents downregulation of the m6A peak and downregulation of mRNA expression. (**B**) Number of DMGs on different chromosomes. (**C**) Chromosomal view of DMGs. (**D**) The top 20 significantly enriched GO terms of the DMGs. (**E**) The top 20 enriched KEGG pathways of DMGs.

Based on the integrated analysis of DMMGs and differentially expressed genes (DEGs), all 111 differentially m6A-methylated and expressed genes (DMGs) were classified into four groups: 14 DMGs showed hypermethylation and upregulation (hyper-up), 13 DMGs exhibited hypomethylation and downregulation (hypo-down), 73 DMGs displayed hypermethylation and downregulation (hyper-down), and 11 DMGs demonstrated hypomethylation and upregulation (hypo-up). The further categorization of these DMGs revealed their m6A methylation positions: 62 DMGs were methylated at exonic regions, 3 DMGs were methylated at ncRNA_exonic regions, 32 DMGs were methylated at 3′ UTR regions, and 14 DMGs were methylated at 5′ UTR regions (Figure 4A). These DMGs were predominantly distributed on chromosomes 3, 2, 10, and 1, with chromosome 2 exhibiting a distribution in exonic, ncRNA_exonic, 3′ UTR, and 5′ UTR regions (Figure 4B,C). This might be attributed to the strong influence of the *Myostatin* gene located on chromosome 2, which could affect other genes in close spatial proximity on this chromosome. Subsequent GO and KEGG analyses highlighted the significant enrichment of genes exhibiting both m6A methylation changes and expression changes in several crucial biological processes and pathways, including the microtubule-associated complex, myosin binding, voltage-gated sodium channel complex, voltage-gated sodium channel activity, and Wnt signaling pathway (Figure 4D,E). These results suggest that m6A methylation may play an important role in regulating the function of important pathways, such as microtubule-associated complexes, myosin binding, and voltage-gated sodium channels.

### 2.5. Network Analysis of Differentially m6A-Methylated and Expressed Genes

**Figure 5:**
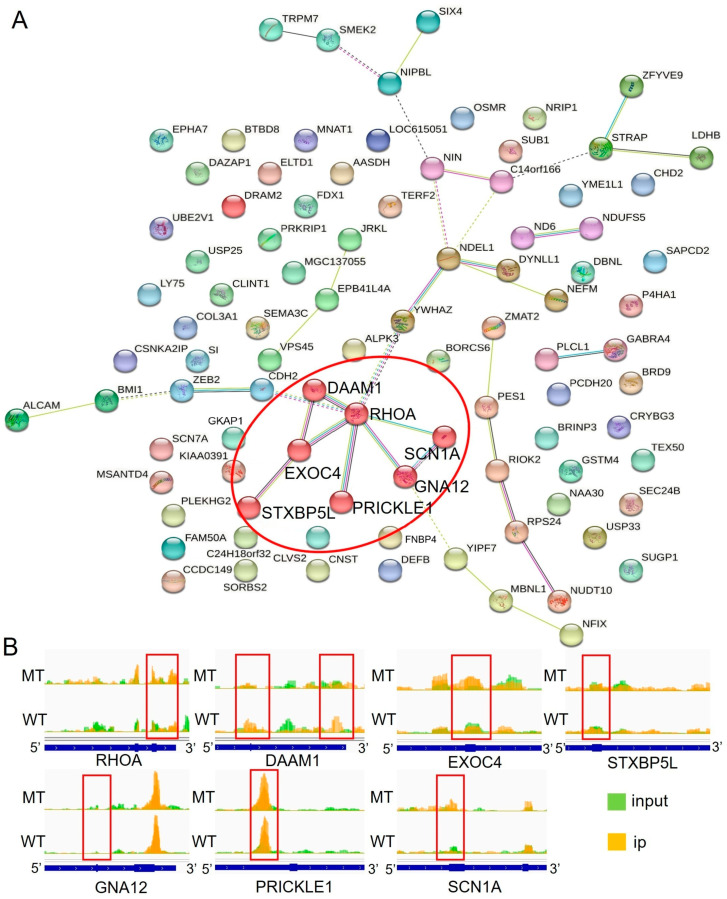
The *RHOA* gene is a key gene in the network of DMGs. (**A**) STRING network analysis of 111 DMGs in the MCL cluster. Inflation parameter = 3. (**B**) Visualization analysis of m6A peaks in the mRNAs of Cluster 1.

To further identify the key genes influencing sperm characteristics, a STRING network analysis was conducted on the MCL clustering of 111 DMGs. This analysis revealed 13 different clusters, with Cluster 1 containing the most genes and a total of seven genes (highlighted as red nodes in Figure 5A). The genes in Cluster 1 included *RHOA*, *DAAM1*, *EXOC4*, *GNA12*, *PRICKLE1*, *SCN1A*, and *STXBP5L*. Table 1 provides a list of the genes within Cluster 1 and their differences between the MT and WT groups. Interestingly, the regions of significant m6A differences in *RHOA*, *DAAM1*, and *GNA12* were identified in the 3′ UTR, while the regions of significant m6A differences in *EXOC4*, *STXBP5L*, and *SCN1A* were found in the exonic regions. Only *PRICKLE1* exhibited m6A modification in the 5′ UTR (Figure 5B). With the exception of *DAAM1*, the m6A methylation of genes in Cluster 1 showed the opposite relationship with mRNA expression levels.

*RHOA* is widely distributed in the head and flagellum of animal spermatozoa and plays an important role in energy acquisition [19]. Our results indicate that the *RHOA* gene is located at the core of the differential gene network, suggesting that m6A modification and the gene expression of *RHOA* in spermatozoa affect the regulation of microtubule synthesis, which may in turn affect sperm motility.

### 2.6. Semen Characteristics, Sperm Motility, and Kinetic Parameters of Movement

To elucidate whether m6A differences affect the basic biological functions of spermatozoa, we conducted further studies on sperm quality. The quality of fresh semen, including ejaculate volume, density, fresh sperm motility, and motion parameters, was evaluated. There were no significant differences in semen volume or sperm count. Fresh sperm motility differed between MT and WT bulls (Table 2). Additionally, the curvilinear line velocity (VCL), straight line velocity (VSL), average path velocity (VAP), amplitude of lateral head displacement (ALH), linearity (LIN), wobble (WOB), straightness (SRT), and beat-cross frequency (BCF) of the MT bull sperm did not significantly change (Table 2).

**Figure 6:**
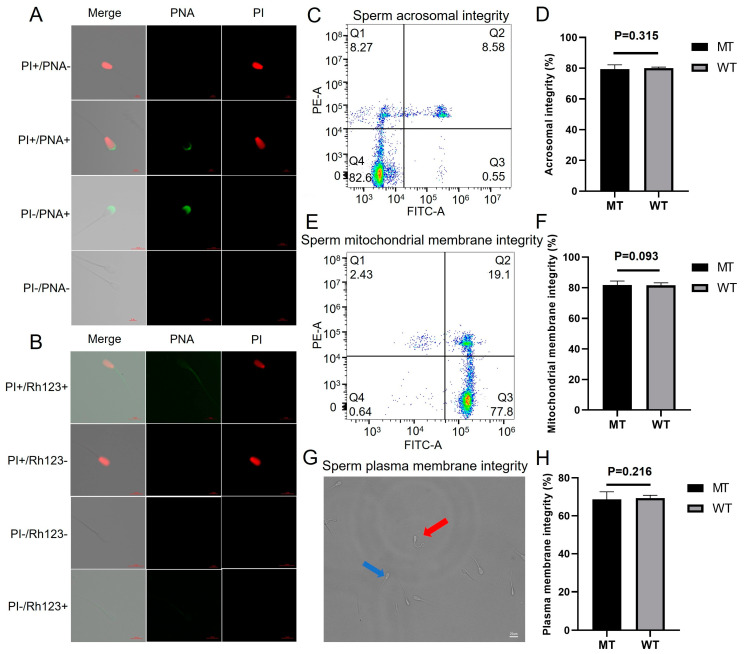
Results of sperm substructural integrity. (**A**) Sperm were analyzed using the acrosome-specific probe Annexin V-FITC/PI. (**B**) Sperm were analyzed using the mitochondrial membrane-specific probe Rh123/PI. (**C**) Flow cytometry plots of acrosomal integrity. (**D**) Histogram of acrosome integrity. (**E**) Flow cytometry plots showing mitochondrial membrane integrity. (**F**) Histogram of mitochondrial membrane integrity. (**G**) Observation of sperm plasma membrane integrity in the hypo-osmotic swelling test (red arrows indicate sperm cells with intact plasma membranes, blue arrows indicate sperm cells with damaged membranes). (**H**) Histogram of plasma membrane integrity.

A further investigation of the morphological features of bull sperm, including the acrosome, plasma membrane, and mitochondrial membrane integrity, was conducted. As shown in Figure 6A, the thawed sperm samples were stained using an Annexin V-FITC/propidium iodide (PI) staining kit, and four subgroups were identified: a subgroup of dead sperm cells with intact acrosome membranes (PI+/PNA-, Q1), a subgroup of dead sperm cells with damaged acrosome membranes (PI+/PNA+, Q2), a subgroup of live sperm cells with damaged acrosome membranes (PI-/PNA+, Q3), and a subgroup of live sperm cells with intact acrosome membranes (PI-/PNA-, Q4). The acrosome integrity rate was analyzed using flow cytometry, and there was no significant difference between the MT group (79.33 ± 2.4%) and the WT group (80.03 ± 0.5%) (*p* = 0.315, Figure 6C,D).

Similarly, the colocalization analysis of MT and WT bull sperm using rhodamine-123 (Rh123) and PI dyes revealed four different scenarios: a subgroup of dead sperm cells with a damaged mitochondrial membrane (PI+/Rh123-, Q1), a subgroup of dead sperm cells with an intact mitochondrial membrane (PI+/Rh123+, Q2), a subgroup of live sperm cells with an intact mitochondrial membrane (PI-/Rh123+, Q3), and a subgroup of live sperm cells with a damaged mitochondrial membrane (PI-/Rh123-, Q4) (Figure 6B). Flow cytometry analysis revealed no significant difference in mitochondrial membrane integrity between the MT and WT groups (83.17 ± 0.5% vs. 79.77 ± 1.6%, *p* = 0.093, Figure 6E,F).

A hypo-osmotic swelling test was performed to measure plasma membrane integrity, and the results revealed no significant difference in the membrane integrity of frozen–thawed sperm between the MT and WT groups (68.80 ± 3.4% vs. 69.55 ± 1.2%, *p* = 0.216, Figure 6G,H). These findings indicate that m6A differences do not significantly affect semen characteristics, sperm motility, or the kinetic parameters of movement.

## 3. Discussion

N6-methyladenosine (m6A) is a crucial methylation modification found in mRNA, serving as a significant epigenetic marker in mammals. The levels of m6A in RNA are dynamically regulated by methyltransferases, such as METTL3 and METTL14 [20], decreased by demethylases, including FTO and ALKBH5 [21], and m6A-binding proteins (readers), which recognize and mediate its functions. This modification has been identified across various organisms, such as viruses, yeast, plants, and animals, including humans [22,23]. The dynamic regulation of m6A modification in mammalian mRNAs introduces a novel epigenetic marker with multifaceted roles in fundamental biological processes including lipid metabolism, spermatogenesis, development, carcinogenesis, and stem cell renewal, as well as other processes yet to be explored [24]. In this study, the overall levels of m6A modifications in the spermatozoa of MT and WT bulls showed no significance and were predominantly located in the CDS region and the 3′ UTR. The differentially expressed m6A genes were found to affect mainly the microtubule-associated complex, myosin binding, voltage-gated sodium channel complex, voltage-gated sodium channel activity, and Wnt signaling pathway. *RHOA*, *DAAM1*, *EXOC4*, *GNA12*, *PRICKLE1*, *SCN1A*, and *STXBP5L* were identified as the seven most crucial DMGs, with *RHOA* being the core gene. Although alterations in m6A modifications may indicate that the *Myostatin* gene affects sperm morphology and motility, no significant changes were observed in sperm viability, acrosome integrity, mitochondrial membrane integrity, or plasma membrane integrity. Further studies are required to investigate the implications of these modifications on other sperm functions.

The primary function of m6A modification is to regulate mRNA stability [25]. This modification can influence the RNA secondary structure and facilitate microRNA target recognition, thereby modulating mRNA stability [26]. In the cytoplasm, m6A-marked mRNAs are recognized by YTHDF2, which promotes mRNA degradation [27]. In the nucleus, m6A modifications regulate RNA splicing and export, contributing to the control of gene expression. Moreover, m6A is implicated in crosstalk with DNA methylation, highlighting its multifaceted role in gene regulation [28]. m6A modification predominantly occurs on conserved motifs such as RRACH (R = G or A, and H = A, C, or U), which are enriched near the stop codon and the 3′ UTR of mRNA [29]. Our experimental investigation revealed noticeable enrichment of the RRACH motif in the mRNA m6A modification profiles of both MT and WT bull sperm. These findings align with previous research suggesting that the presence of the *Myostatin* gene does not influence the enrichment of m6A modification motifs in sperm mRNA.

During periods of body weight loss, there was no significant impact on sperm DNA 5-methylcytosine methylation levels. However, RNA m6A methylation levels significantly decreased. Conversely, during periods of body weight gain, both DNA 5-methylcytosine and RNA m6A methylation levels showed significant increases [30]. In line with these findings, our study obtained similar results. Since the MT bulls had a greater body weight than the WT bulls at the same age, their sperm exhibited increased m6A modification (Figure 2D,E).

Testosterone, a key steroid hormone, plays a critical role in spermatogenesis [31]. Alterations in steroid hormone production can significantly influence sperm development [32]. Steroid hormones, derived from cholesterol, can be divided into two main groups: sex hormones and adrenocorticotropic hormones [33]. Due to their lipid solubility, these hormones are derived from cholesterol and possess lipid solubility, allowing them to traverse the plasma membrane and bind to intracellular receptors, known as nuclear receptors (NRs), to regulate gene expression. The synthesis of steroid hormones primarily occurs in the adrenal cortex, gonads (testes and ovaries), brain, placenta, and adipose tissue. Two major enzyme classes, cytochrome P450 enzymes (CYPs) and hydroxysteroid dehydrogenases (HSDs), are involved in this process [34]. The first step in steroid hormone synthesis is the removal of six carbons from the side chain of cholesterol to produce pregnenolone. This is the rate-limiting step in the pathway and consists of a three-step reaction. All of these reactions are catalyzed in humans by cholesterol-20,22-desmolase (also known as CYP11A or P450ssc), a cholesterol side-chain cleaving enzyme [35]. This enzyme binds to the inner membrane of the mitochondria in all steroidogenic tissues, so cholesterol needs to be transported to the mitochondria first. The translocation process is mediated by the rapid regulation of steroid synthesis on the outer membrane, which is the rate-limiting step of this process [36]. In this study, DMMGs were enriched in the steroid hormone biosynthesis and drug metabolism-cytochrome P450 pathways (Figure 3). This suggests that m6A methylations in MT bull sperm may alter CYP enzymes, impacting steroid hormone biosynthesis and potentially affecting mitochondrial functions during spermatogenesis.

Sperm motility and structural integrity are crucial for successful fertilization in vivo. Disruptions in the assembly of the axoneme and the surrounding structures in the sperm flagellum can result in reproductive issues [37]. In this study, although differences in the m6A peaks were observed, the overall pattern remained largely unchanged (Figure 1B,C and Figure 2A). This may explain why sperm characteristics such as sperm motility in MT bulls were not significantly different from those in WT bulls. Rho GTPases, members of the Ras superfamily with 25% homology with Ras, are essential regulators of cellular functions. Among them, RHOA is widely distributed in the head and flagellum of animal sperm and plays a key role in energy acquisition [38]. Previous studies have shown that the exogenous addition of recombinant RHOA protein improves semen quality and maintains subcellular structural homeostasis in rooster sperm after cryopreservation and thawing. These effects are mediated via the Rho/ROCK pathway, which regulates the dynamic reorganization of the actin cytoskeleton through modulating cofilin phosphorylation, thereby improving sperm viability, survival, linearity, linear velocity, and acrosome integrity following freeze–thaw procedures [39]. Furthermore, recent findings have revealed that the m6A modification of *RHOA* can be inherited by offspring, contributing to hippocampal neuron aging and cognitive impairments induced by environmental factors such as cadmium exposure and a high-fat diet [40]. These insights underscore the pivotal role of *RHOA* and its m6A modification in sperm biology. However, further investigations are needed to unravel the precise mechanisms underlying the influence of *RHOA* and other related factors on sperm function and male fertility [41].

A previous study on transcriptome-wide m6A modifications in male mosquito reproductive tissues and sperm revealed a significant abundance of m6A methylation in both RNA samples, with higher levels of m6A methylation detected in sperm. Differentially expressed between m6A-modified and unmodified transcripts identified several m6A-associated regulatory pathways related to sperm tail formation, including genes involved in microtubule and ATP synthesis, which are important for sperm motility [42]. The tail of the spermatozoon plays a crucial role in sperm motility, generating propulsive force through flagellar movement. A high concentration of mitochondria is present in sperm, and ATP produced through mitochondrial metabolism serves as the primary energy source for sperm motility, activating the flagellar system. Increased integrity of the mitochondrial membrane may contribute to enhancements in sperm motility parameters. In our study, no significant changes were observed in the motion parameters or mitochondrial membrane integrity of sperm from MT bulls (Figure 6). While differentially methylated genes were enriched in pathways such as microtubule-associated complex and myosin binding, these effects did not directly influence sperm motion characteristics or morphology. The potential impacts of these pathways on sperm function require further investigation.

## 4. Materials and Methods

### 4.1. Animals

The *Myostatin* gene-edited cattle were bred and genotyped within our laboratory [2]. For semen characterization analysis, we selected three MT bulls and three WT bulls to minimize variables. Both groups were raised and managed under consistent conditions on the same farm. A dedicated team was responsible for their care, ensuring uniform diets and free access to water. All animal experiments followed the Bioethics Committee of Inner Mongolia University (IMU-CATTLE-2022-061) regulations.

### 4.2. Semen Cryopreservation and Sperm Sample Collection

Bull semen was collected, evaluated, and cryopreserved following the protocol outlined in our recent study [43],. Semen specimens were obtained utilizing the artificial vaginal method. The characteristics of the sperm were measured using an SCA automated sperm analyzer (024905, IMV). The analyzed sperm characteristics included MOT (motility), VCL (curvilinear velocity), VSL (straight line velocity), VAP (average path velocity), ALH (amplitude of lateral head displacement), LIN (linearity of movement), WOB (wobbling index), SRT (straightness), and BCF (beat-cross frequency).

### 4.3. Sperm Quality Analysis

The sperm plasma membrane integrity was determined through a hypo-osmotic swelling test. The integrity of the sperm mitochondria was evaluated by staining with rhodamine-123 (Rh123) and propidium iodide (PI), followed by analysis via flow cytometry. The integrity of the sperm acrosome was assessed using Peanut Agglutinin-FITC (FITC-PNA) and PI staining, followed by analysis via flow cytometry. The specific experimental procedures were conducted according to our recent study [43]. During flow cytometry analysis, 20,000 cells were selected for each experiment, and three replicates were performed for each semen sample.

### 4.4. RNA Isolation, Library Construction, and Sequencing

Total RNA was isolated from the sperm of 2 MT bulls and 2 WT bulls using TRIzol reagent according to the manufacturer’s instructions (Invitrogen, Carlsbad, CA, USA). The RNA was quantified for purity and quantity using NanoDrop ND-1000 (NanoDrop, Wilmington, DE, USA), and its integrity was assessed by Bioanalyzer 2100 (Agilent, Santa Clara, CA, USA) with an RNA integrity number (RIN) > 7.0. Total RNA (>1 μg) was fragmented into small pieces at 94 °C for 5 min using the Magnesium RNA Fragmentation Module (NEB, cat.e6150, Ipswich, MA, USA) and further incubated with m6A antibody-dynabead compounds. The IP RNA was reverse transcribed using SMART Scribe™ Reverse Transcriptase (CloneTech, cat. 634414, Shiga, Japan) to generate first-strand cDNA. The cDNA was then used for adapter ligation and the synthesis of second-strand DNAs via polymerase chain reaction (PCR) using the following conditions: initial denaturation at 94 °C for 1 min; denaturation at 98 °C for 15 s, annealing at 55 °C for 15 s, and extension at 68 °C for 30 s, followed by a final extension at 68 °C for 2 min for 5 cycles. The amplified DNA-seq library was purified by immobilization onto pure beads, and cDNA sequences originating from rRNA reverse transcription were cut by ZapR v2 and R-Probes v2 (for mammals) under incubation at 72 °C for 2 min, 4 °C for 2 min, 37 °C for 1 h, and 72 °C for 10 min with subsequent cooling at 4 °C. Finally, the library was subjected to a second round of PCR (12–16 cycles) that was consistent with the first round of PCR. The library was sequenced via 2 × 150 bp paired-end sequencing (PE150) on an Illumina NovaSeq 6000 (Illumina, San Diego, CA, USA) following the vendor’s recommended protocol.

### 4.5. Data Analysis

Raw sequencing data in fastq format were subjected to quality control using fastp (v0.19.4) [44] with default parameters. This included adapter trimming, removal of duplicate sequences, and filtering of low-quality reads. The resulting CleanData were retained for subsequent analysis. CleanData were aligned to the *Bos taurus* reference genome (v107) using HISAT2 (v2.0.4) [45], and BAM files were generated for downstream analysis. The exomePeak package [46] in R was used to identify and analyze chromatin accessibility peaks from the BAM files. Differential peak analysis was performed to identify significant differences between IP samples and input samples (Tables S1 and S2). ANNOVAR [47] was used for annotating the identified peaks with genomic features such as gene names, functional regions, and regulatory elements. Motif analysis to identify enriched DNA sequence motifs within the peaks was performed using MEME2 (v5.3.3) [48] and HOMER (v4.10). StringTie (v2.1.2) [49] was used for genome assembly and quantification of gene expression levels via the FPKM method. Differential expression analysis was performed using the edgeR package (v4.1) [50] in R, considering genes with a fold change (FC) ≥ 2 or ≤0.5 and a *p* < 0.05 to be significantly differentially expressed. DAVID Bioinformatics Resources (https://david.ncifcrf.gov; v6.8) were utilized for gene ontology (GO) analysis and domain annotation. The STRING database (https://string-db.org, v11.5) was used to determine gene–gene interaction relationships.

All data are presented as the mean ± standard deviation (SD). Statistical analysis (*t* test) was performed using GraphPad Prism v9.5, with *p* < 0.05 considered to indicate statistical significance and marked with *.

## 5. Conclusions

In this study, we provided a comprehensive map of m6A methylations in MT bull spermatozoa. The differential m6A methylation genes affected the sperm cell membrane and altered the G protein-coupled receptor signaling pathway. We also found that the *RHOA* gene is the core gene of the differentially m6A-methylated and expressed genes network, which demonstrates the potential function of *Myostatin* gene editing in affecting sperm morphology and motility. However, through analysis, it was found that m6A methylation levels did not seem to significantly affect ejaculate volume, density, fresh sperm viability, or motility parameters. This study provides new insights into the functional role of m6A modification and gene expression in bull spermatozoa. These findings reveal potential genetic factors affecting sperm characteristics that need to be further analyzed, offering valuable insights into the production of high-quality MT cattle.

## Figures and Tables

**Table 1:** Information on the DMGs in Cluster 1.

Gene	m6A Diff_Log^2^	Gene Diff_Log^2^	Region
RHOA	2.69	−1.52	3′ UTR
DAAM1	−3.49	−1.53	3′ UTR
EXOC4	1.7	−1.31	exonic
STXBP5L	1.94	−1.39	exonic
GNA12	−1.55	1.52	3′ UTR
PRICKLE1	1.11	−1.37	5′ UTR
SCN1A	2.91	−1.22	exonic

**Table 2:** Statistical analysis of Mongolian cattle biochemical parameters in different regions.

Semen (Sperm) Parameters	MT (Mean ± SD)	WT (Mean ± SD)	*p* Value
Semen volume (mL)	5.6 ± 1.0	4.8 ± 1.2	0.346
Sperm count (×10^9^/mL)	1591.67 ± 180.10	1319 ± 198.67	0.153
MOT (%)	80.87 ± 5.2	78.77 ± 6.1	0.675
VCL (μm/s)	86.05 ± 2.35	84.59 ± 3.05	0.542
VSL (μm/s)	52.27 ± 2.40	50.97 ± 3.54	0.625
VAP (μm/s)	56.83 ± 1.08	54.52 ± 1.32	0.212
ALH (μm)	3.19 ± 0.05	3.19 ± 0.03	0.837
LIN (%)	60.74 ± 1.20	60.25 ± 1.10	0.609
WOB (%)	66.04 ± 1.28	64.45 ± 1.21	0.183
SRT (%)	91.98 ± 2.05	93.49 ± 3.34	0.540
BCF (Hz)	5.2 ± 1.3	5.0 ± 1.6	0.979

MOT (motility), VCL (curvilinear line velocity), VSL (straight line velocity), VAP (average path velocity), ALH (amplitude of lateral head displacement), LIN (linearity, VSL/VCL%), WOB (wobble, VAP/VCL %), SRT (straightness, VSL/VAP %), BCF (beat-cross frequency).

## Data Availability

The original contributions presented in the study are included in the article/Supplementary Materials, and further inquiries can be directed to the corresponding author/s.

## Supplementary Materials

The following supporting information can be downloaded at: https://www.mdpi.com/article/10.3390/ijms26020591/s1.
