# Supplementary material for: Loss of Myostatin Affects m6A Modification but Not Semen Characteristics in Bull Spermatozoa
Source: Int J Mol Sci. 2025 Jan 12;26(2):591. doi: 10.3390/ijms26020591 (PMC11766052; doi:10.3390/ijms26020591)
Supplement: Supplementary file 1 [file ijms-26-00591-s001.zip › ijms-3398579-supplementary.pdf]

Supplementary data

Table S1 Summary of the quality control statistics for the sequencing reads

Sample	Raw_Reads	Valid_Reads	Valid%	Q20%	Q30%	GC%
MT1_IP	51,197,684	50,291,340	95.98	96.86	91.86	38.89
MT2_IP	54,500,886	53,519,414	95.98	96.99	92.07	39.29
WT1_input	53,743,388	53,177,110	94.42	97.8	93.72	44.67
MT2_input	55,146,064	54,563,258	94.35	97.78	93.71	45.77

Table S2 Reference genome comparison

Sample	Valid reads	Mapped reads	Unique Mapped reads	Reads map to sense strand	Reads map to antisense strand	Non-splice reads
WT1_IP	51,178,894	44,822,027 (87.58%)	33,080,232 (64.64%)	22,303,470 (43.58%)	22,160,097 (43.30%)	44,283,408 (86.53%)
WT2_IP	50,197,866	44,403,922 (88.46%)	32,040,409 (63.83%)	22,107,504 (44.04%)	21,967,126 (43.76%)	43,907,711 (87.47%)
MT1_IP	50,073,892	42,018,436 (83.91%)	31,089,891 (62.09%)	20,901,447 (41.74%)	20,758,123 (41.45%)	41,517,027 (82.91%)
MT2_IP	53,130,772	45,289,875 (85.24%)	33,596,880 (63.23%)	22,523,893 (42.39%)	22,358,701 (42.08%)	44,591,866 (83.93%)
WT1_input	53,275,676	44,252,262 (83.06%)	31,516,816 (59.16%)	21,832,560 (40.98%)	21,552,092 (40.45%)	43,060,763 (80.83%)
WT2_input	53,071,982	44,278,179 (83.43%)	31,790,923 (59.90%)	21,800,514 (41.08%)	21,553,833 (40.61%)	43,002,602 (81.03%)
MT1_input	54,068,864	41,872,062 (77.44%)	30,063,824 (55.60%)	20,482,133 (37.88%)	20,234,068 (37.42%)	40,275,990 (74.49%)
MT2_input	47,318,316	36,404,886 (76.94%)	26,152,541 (55.27%)	17,658,679 (37.32%)	17,472,434 (36.93%)	34,654,703 (73.24%)
